# Follicular Lesions with Papillary Nuclear Characteristics: Differences in Chromatin Detected by Computerized Image Analysis

**DOI:** 10.20945/2359-3997000000282

**Published:** 2020-08-24

**Authors:** Bárbara Parente Coelho, Flávia de Oliveira Valentim, Hélio Amante Miot, Danilo Takeshi Abe Jaune, Caroline Yuki Hayashi, Cristiano Claudino de Oliveira, Mariângela de Alencar Marques, José Vicente Tagliarini, Emanuel Celice Castilho, Paula Soares, Gláucia Maria Ferreira da Silva Mazeto

**Affiliations:** 1 Universidade Estadual de São Paulo Faculdade de Medicina de Botucatu Departamento de Cirurgia e Ortopedia Botucatu SP Brasil Departamento de Cirurgia e Ortopedia, Faculdade de Medicina de Botucatu, Universidade Estadual de São Paulo (Unesp), Botucatu, SP, Brasil; 2 Universidade Estadual de São Paulo Faculdade de Medicina de Botucatu Departamento de Dermatologia Botucatu SP Brasil Departamento de Dermatologia, Faculdade de Medicina de Botucatu, Universidade Estadual de São Paulo (Unesp), Botucatu, SP, Brasil; 3 Universidade Estadual de São Paulo Faculdade de Medicina de Botucatu Departamento de Medicina Interna Botucatu SP Brasil Departamento de Medicina Interna, Faculdade de Medicina de Botucatu, Universidade Estadual de São Paulo (Unesp), Botucatu, SP, Brasil; 4 Universidade Estadual de São Paulo Faculdade de Medicina de Botucatu Departamento de Patologia Botucatu SP Brasil Departamento de Patologia, Faculdade de Medicina de Botucatu, Universidade Estadual de São Paulo (Unesp), Botucatu, SP, Brasil; 5 Universidade Estadual de São Paulo Faculdade de Medicina de Botucatu Departamento de Otorrinolaringologia e Cirurgia de Cabeça e Pescoço Botucatu SP Brasil Departamento de Otorrinolaringologia e Cirurgia de Cabeça e Pescoço, Faculdade de Medicina de Botucatu, Universidade Estadual de São Paulo (Unesp), Botucatu, SP, Brasil; 6 Universidade do Porto Instituto de Investigação e Inovação em Saúde (i3S) Porto Portugal Instituto de Investigação e Inovação em Saúde (i3S), Universidade do Porto, Porto, Portugal; 7 Universidade do Porto Instituto de Patologia Molecular e Imunologia Grupo de Sinalização e Metabolismo do Câncer Porto Portugal Grupo de Sinalização e Metabolismo do Câncer, Instituto de Patologia Molecular e Imunologia da Universidade do Porto (IPATIMUP), Porto, Portugal; 8 Universidade do Porto Faculdade de Medicina Departamento de Patologia Porto Portugal Departamento de Patologia, Faculdade de Medicina da Universidade do Porto, Porto, Portugal

**Keywords:** Carcinoma, Papillary, Follicular, Cell nucleus, Histology, Thyroid neoplasms

## Abstract

**Objective:**

Follicular lesions of the thyroid with papillary carcinoma nuclear characteristics are classified as infiltrative follicular variant of papillary thyroid carcinoma-FVPTC (IFVPTC), encapsulated/well demarcated FVPTC with tumour capsular invasion (IEFVPTC), and the newly described category “non-invasive follicular thyroid neoplasm with papillary-like nuclear features” (NIFTP) formerly known as non-invasive encapsulated FVPTC. This study evaluated whether computerized image analysis can detect nuclear differences between these three tumour subtypes.

**Materials and methods:**

Slides with histological material from 15 cases of NIFTP and 33 cases of FVPTC subtypes (22 IEFVPTC, and 11 IFVPTC) were analyzed using the *Image J* image processing program. Tumour cells were compared for both nuclear morphometry and chromatin textural characteristics.

**Results:**

Nuclei from NIFTP and IFVPTC tumours differed in terms of chromatin textural features (grey intensity): mean (92.37 ± 21.01 *vs* 72.99 ± 14.73, p = 0.02), median (84.93 ± 21.17 *vs* 65.18 ± 17.08, p = 0.02), standard deviation (47.77 ± 9.55 *vs* 39.39 ± 7.18; p = 0.02), and coefficient of variation of standard deviation (19.96 ± 4.01 *vs* 24.75 ± 3.31; p = 0.003). No differences were found in relation to IEFVPTC.

**Conclusion:**

Computerized image analysis revealed differences in nuclear texture between NIFTP and IFVPTC, but not for IEFVPTC.

## INTRODUCTION

Papillary thyroid carcinoma (PTC) is the most common thyroid cancer. There are many histological variants, the most common being the classic and follicular (FVPTC) forms. Diagnoses of the latter form have become more common (1), with two main subtypes: infiltrative (IFVPTC) and encapsulated (2,3), which has also been shown as more frequent (1).

Although FVPTC is generally associated with a better prognosis than classic PTC (4), there are still questions as to the behaviour of its histological subtypes. Encapsulated FVPTC, for example, represents a diagnostic challenge and seems to present its own genetic and evolutive characteristics (5,6), such as different biological behaviour. Recently, a group of experts compared patients with encapsulated/well demarcated FVPTC with tumour capsular invasion (IEFVPTC) against those without vascular and/or tumour capsule invasion (non-invasive); they reported that the non-invasive tumour had a distinct behaviour with very low risk of progression, proposing the name non-invasive follicular thyroid neoplasms with papillary-like nuclear features, or NIFTP (7). Based on these findings, the World Health Organization added NIFTP to their classification list of endocrine tumours, with diagnosis based on very specific histopathological criteria such as well-defined lesion demarcation, absence of capsular of vascular infiltration, and the presence of atypical nuclear features similar to those in PTC (8). Thus, only a very specific subset of non-invasive encapsulated tumours can now be reclassified as NIFTP.

In spite of the fact that this new classification represents an undeniable advance (9), there are situations in which the cited histopathological criteria cannot be fully evaluated, for example due to a lack of tumour material integrity. In fact, in the same way as in follicular adenomas and carcinomas, NIFTP diagnosis depends on detailed evaluation of the whole tumour capsule to exclude invasion (7). Also, the criteria used by different services for the definition of nuclear atypia can present certain heterogeneity (10).

Computerized image analysis of follicular cell nuclei is a proven reproducible, low cost, operator independent method representing a diagnosis support tool which is potentially useful in evaluating thyroid tumours (11–13). Recently, using this method, Valentim and cols. reported high discriminatory power between follicular carcinomas and adenomas and between the latter and FVPTC (14). In this sense, these computer resources could contribute to the differential diagnosis of NIFTP, conferring more objectivity to pathological evaluation. Therefore, this study evaluated whether computerized image analysis can detect nuclear differences between NIFTP and the FVPTC subtypes.

## MATERIALS AND METHODS

This cross-sectional study compared NIFTP, IEFVPTC and IFVPTC tumour subtypes for nuclear morphometry and chromatin textural characteristics. Tumoral histological material from the 48 follicular lesions was reviewed by two experienced pathologists (MEAM/CCO), and was reclassified as NIFTP (n = 15), IEFVPTC (n = 22), or IFVPTC (n = 11) according to recommendations (7,8). Only cases where tumour capsule was completely included in histopathological analysis were included in this study. New sections were cut from the paraffin block samples, mounted on slides, and stained with haematoxylin-eosin (HE).

Images were acquired by the same pathologists (MEAM/CCO) from the HE sections. Areas of the slides most representative of tumours and with a high number of nuclei were photographed using a PANORAMIC MIDI II – 3D camera (Histech, Japan; http://3dhistech.com/pannoramic_midi) at 43X magnification. In general, three or four photographs were taken of each slide and the best photo chosen and randomly numbered for analysis.

**Figure 1:**
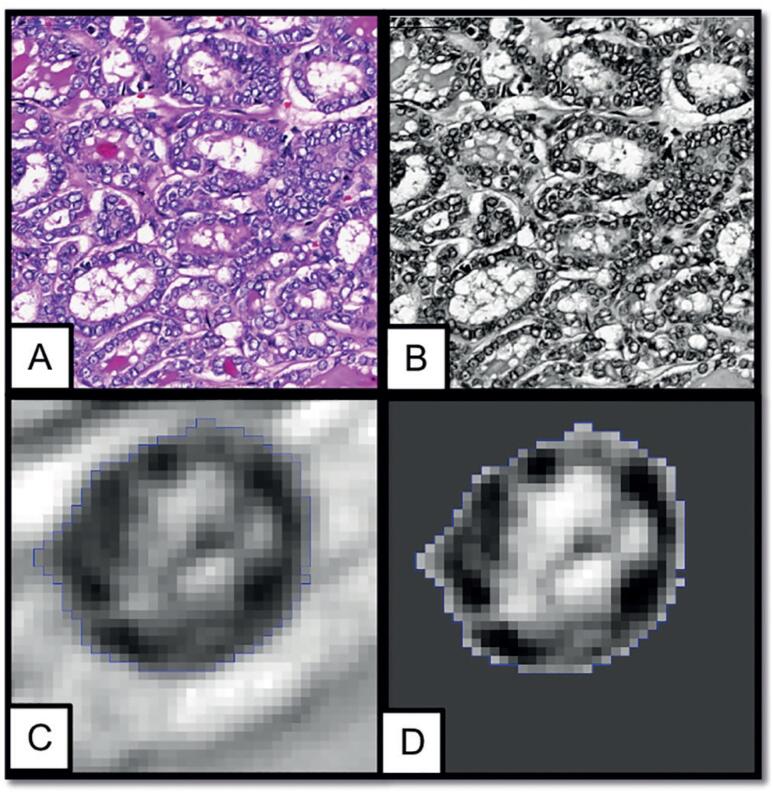
Slide photo exemplifying the morphometric analysis of the histological material of a follicular variant of papillary carcinoma (FVPTC) under analysis by Image J program. The program transforms the millions of tones of the captured color images into 256 tones of grey (grey-scale, 16-bits), considerably reducing the number of tones in the nuclei images, allowing a much clearer evaluation of chromatin compaction level and better homogeneity between samples despite differences in color A: color photo; B: grey-scale photo; C and D: selected nucleus.

Histological images were submitted to nuclear analysis with the help of the *Image J* computer program (15), by two researchers who had no access to the final histopathological diagnosis (blinded analysis). Briefly, the program transforms the millions of colours from the captured colour images into 256 grey tones (16-bit grey-scale), considerably reducing the number of tones in the nuclei images, allowing a much clearer evaluation of chromatin compaction level and better homogeneity between samples despite differences in colour (13). Manual selection and individual analysis of nuclei was performed from the grey-scale images (Figure 1), those with nuclei clustering and superimposition were excluded. After nuclear selection, the *Image J* program evaluated a series of morphometric and textural parameters (16). Nuclear morphometric evaluation included the following parameters: area; perimeter; circularity; larger diameter (Feret); area per Feret and perimeter/area indexes; and larger/smaller diameter (Aspect Ratio, AR), perimeter/area (P/A), and area/Feret ratios. The morphometric parameters, expressed in pixels, were converted to microns (µm). Evaluation of textural chromatin characteristics included: mean, median and standard deviation (STDEV) of grey intensity (expressed in a scale of 256 shades of grey, in which higher numbers indicate lighter nuclei: 0 = black; 255 = white); roughness (RA); regularity of nuclear membrane (Round); solidity; fractal dimension (Fractal); entropy; and grey intensity/area ratio. Secondary indicators relative to evaluated parameters (coefficients of variation) were also calculated: coefficients of variation (CV) for Mean intensity (CV-Mean intensity), STDEV (CV-STDEV), and Median intensity (CV-Median intensity).

The minimum number of nuclei to be studied in each photo was obtained from a previous statistical study using a standard error of 5% for each variable, assuring that, from the value obtained, any further increase in number of nuclei would not affect the final result. In this way, we evaluated 60 nuclei per photo, a total of 2,880 in all.

Calculations were performed using a median of 60 nuclear measurements per case. Nuclear parameters were tested for normality using the Shapiro-Wilk test and described as mean and standard deviation or median and quartiles (25% and 75%; p25-p75) according to data normality (17). The three tumour subtypes were initially tested using ANOVA for normal and the Kruskal-Wallis test for non-normal distribution. Parameters showing statistical differences in the comparative tests were submitted to *post-hoc (Sidak)* analysis to test which tumours differed between each other. Significance was considered when p < 0.05 in the bicaudal test.

This study was approved by the local Research Ethics Committee (protocol number 635.459; CAAE number 29089214.1.0000.5411).

## RESULTS

Patients with NIFTP, IEFVPTC and IFVPTC did not differ for age [median (minimum maximum) of 49 (34.5; 61), 52,5 (44.5; 58.25), and 52 (44.5; 53.5) years, respectively; p = 0.85] or gender [13 (86.7%), 22 (100%), and 10 (90.9%) women, respectively; p = 0.15]. Mean tumour diameter (±standard deviation) was greater in IEFVPTC (2.57 ± 1.67 cm) than IFVPTC (1.25 ± 0.89 cm; p = 0.03), however NIFTP did not show difference when compared to the other two groups (1.86 ± 1.29 cm; p > 0.05).

Comparing the three tumour subtypes (Table 1), NIFTP and IFVPTC showed significant differences in the texture parameters of mean grey intensity [92.37 ± 21.01 *versus* (*vs*) 72.99 ± 14.73; p = 0.02), median grey intensity (84.93 ± 21.17 *vs* 65.18 ± 17.08; p = 0.02), STDEV (47.77 ± 9.55 *vs* 39.39 ± 7.18; p = 0.02), and CV-STDEV (19.96 ± 4.01 *vs* 24.75 ± 3.31; p = 0.003), indicating that the former presents clearer nuclei with more heterogenous colouration than the latter. IEFVPTC showed no difference in any nuclear parameter in relation to NIFTP or IFVPTC.

**Table 1:** Morphometric and textural parameters of encapsulated/well demarcated follicular variant of papillary thyroid carcinoma with tumour capsular invasion, infiltrative follicular variant of papillary thyroid carcinoma, and noninvasive follicular thyroid neoplasms with papillary-like nuclear features

Parameter	Tumors			P
	NIFTP (n = 15)	IEFVPTC (n = 22)	IFVPTC (n = 11)	
Area (µm²)*	57.38 ± 15.98	64.46 ± 18.95	53.61 ± 14.24	0.58
Mean gray intensity*†	92.37 ± 21.01^b^	81.72 ± 21.3	72.99 ± 14.73^a^	**0.02**
STDEV*†	47.77 ± 9.55^b^	44.51 ± 8.22	39.39 ± 7.18^a^	**0.02**
Perimeter (µm)*	30.86 ± 4.65	31.7 ± 4.95	29.57 ± 3.34	0.48
Circularity**	0.77 (0.69; 0.89)	0.84 (0.74; 0,87)	0.81 (0.71; 0.87)	0.52
Feret (µm)*	10.81 ± 1.65	11.18 ± 1.58	10.56 ± 1.0	0.68
Median gray intensity*†	84.93 ± 21.17^b^	73.05 ± 20.8	65.18 ± 17.08^a^	**0.02**
AR**	1.37 (1.24; 1.43)	1.31 (1.26; 1.36)	1.33 (1.27; 1.59)	0.73
Roundness**	0.73 (0.71; 0.81)	0.76 (0.74; 0.8)	0.76 (0.63; 0.79)	0.76
Solidity**	0.9 (0.88; 0.95)	0.93 (0.9; 0.95)	0.92 (0.89; 0.94)	0.37
Fractal*	2.46 ± 0.04	2.46 ± 0.03	2.44 ± 0.04	0.28
Entropy*	4.62 ± 0.62	4.76 ± 0.48	4.51 ± 0.89	0.67
Perimeter/Area (µm^−1^)**	0.5 (0.48; 0.68)	0.49 (0.46; 0.55)	0.56 (0.48; 0.66)	0.18
RA*	99.49 ± 19.16^a^	110.15 ± 21.07	115.66 ± 20.47^b^	0.05
Area/Feret (µm)**	5.63 (4.29; 5.92)	5.9 (4.91; 6.33)	4.77 (4.58; 5.97)	0.18
Gray intensity/Area (units/µm^−2^)**	0.14 (0.12; 0.18)	0.12 (0.11; 0.15)	0.12 (0.12; 0.15)	0.15
CV – Mean intensity**	21.45 (19.53; 24.27)	21.95 (20.31; 26.46)	22.63 (20.47; 26.26)	0.45
CV-STDEV*	19.96 ± 4.01^a^	22.34 ± 4.08	24.75 ± 3.31b	**0.003**
CV – Median Intensity**	26.86 (23.22; 30.76)	26.63 (24.25; 36.01)	28.13 (25.62; 33.75)	0.46

* Mean ± standard deviation.

** Median (percentile 25; percentile 75).

Statistical test: ANOVA and Kruskal Wallis, complemented by the Multiple Comparisons test.

† Expressed in a scale of 256 shades of grey, in which higher numbers mean lighter nuclei (0 = black; 255 = white).

Different letters mean statistical difference (bold indicates significant differences between a and b; p < 0.05). AR: *Aspect Ratio*; CV: coefficient of variation; IEFVPTC: encapsulated/well demarcated follicular variant of papillary carcinoma with tumour capsular invasion; IFVPTC: infiltrative follicular variant of papillary carcinoma; NIFTP: noninvasive follicular thyroid neoplasms with papillary-like nuclear features; RA: roughness; STDEV: standard deviation of gray intensity; µm: micrometer.

## DISCUSSION

In this study, we observed significant differences between NIFTP and IFVPTC. In general, the differential between infiltrative (IFVPTC) and encapsulated FVPTC depends on evaluating a series of both qualitive and quantitative histological parameters. Differentiation between the encapsulated lesions (NIFTP and IEFVPTC), in turn, would be based on more complex morphological criteria, i.e., the presence of vascular and capsular invasion, quantification of mitotic activity, the presence of papillary structures, and of solid/trabecular/insular growth pattern, as well as using nuclear scores (7). All this analysis requires highly specialized training and meticulous evaluation, as well as the adequate supply and preparation of histological material. In this sense, tools which can help in the diagnostic process would be very welcome.

We observed nuclear textural differences between NIFTP and IFVPTC, indicating that the former presents clearer nuclei with more heterogenous colouration than the latter. Chromatin clearing is a prominent feature in FVPTC and can be present in any of the three tumour subtypes, but, as with other nuclear characteristics, it is not often used to compare them. However, the more aggressive tumour would be expected to exhibit more prominent nuclear atypia, including nucleoplasm clearing. Thus, our findings remain to be clarified. Other features have been cited as criteria for diagnosing NIFTP (7). Considering these additional parameters, diagnosing this subtype depends on the availability, integrity, and evaluation of all the tumour material which is not always possible. In this way, the technique used here, evaluating only the cell nucleus, could contribute to differential diagnosis between NIFTP and IFVPTC, particularly in situations where all the included histological material was not sufficient.

The findings of the present study were as expected, since assuming a progressive malignancy grading, with NIFTP (the best prognosis tumour) and IFVPTC (the worst), it would be plausible that both presented different nuclear characteristics. On the other hand, no significant differences were found between the encapsulated variants of FVPTC with and without invasive features (NIFTP and IEFVPTC). The reasons for these findings still require clarification. It is true that the morphological differentiation between these two lesions is more complex, as it involves analysis of the presence or lack of tumour capsular invasion. As computerized image analysis of the nuclear pattern does not help diagnostic definition in these variants, perhaps the capsular question requires further study for a better biological understanding of these neoplasms. We can hypothesize that nuclear features are influenced by the genetic background as several studies have indicated that NIFTP and IEFVPTC share a higher rate of RAS mutations, at variance with IFVPTC that show more frequently BRAF mutations. Thus, encapsulated, and particularly NIFTP tumours would be closer to the group of follicular neoplasms than to PTC, as in the case of IFVPTC (7,18). In addition, it has been suggested that there is temporal evolution accompanied by genetic mutations which can lead to an initially standard NIFTP tumour becoming an IEFVPTC (7).

Actually, nuclear morphological and textural aspects have been used in diagnostic evaluation of FVPTC subtypes. Similar to our results, Maletta and cols. reported nuclear differences between benign nodules and NIFTP, but not between NIFTP and IEFVPTC where overlapping was observed (19). Perhaps the differences between NIFTP and IEFVPTC are not easily detectable, or so expressive, when only considering nuclear characteristics, and other elements (morphologic, histopathological, genetic) must be included in the equation. It is known that some features such as the presence of nodal metastases and BRAF^V600E^ mutation appear to differentiate these tumours (20). However, it would be desirable that earlier and/or less expensive/complex diagnostic methods were available to differentiate them.

The main limitation of this study is the small number of samples evaluated, which although small, was still sufficient to identify the above reported differences. Despite this limitation, this study showed that computerized image analysis, an objective and reproducible method, could be a valuable tool to identify nuclear differences between IFVPTC, a more aggressive tumour, and NIFTP, a less aggressive lesion. From a practical point of view, as suggested above, the method could be particularly useful in the diagnostic evaluation of neoplasms in which all tumoral tissue is not available for histopathological evaluation.

In conclusion, we verified that a computerized image analysis tool detected nuclear textural differences between NIFTP and IFVPTC, but not between NIFTP and IEFVPTC. Therefore, optimization of this technique, with the integration of additional morphological, genetic or histopathological parameters, could represent a useful complimentary diagnostic tool for evaluating follicular patterned lesions with papillary-like nuclear characteristics. New studies with larger sample numbers are needed to confirm and expand upon the results obtained in this study.

## References

[1] Jung CK, Little MP, Lubin JH, Brenner AV, Wells Jr SA, Sigurdson AJ, et al. The increase in thyroid cancer incidence during the last four decades is accompanied by a high frequency of BRAF mutations and a sharp increase in RAS mutations. J Clin Endocrinol Metab. 2014;99(2):E276-85. 10.1210/jc.2013-2503 PMC3913801 24248188

[2] Rosai J, Carcangiu ML, DeLellis RA. Atlas of Tumor Pathology. Washington, DC: Armed Forces Institute of Pathology; 1993. 343p.

[3] Liu J, Singh B, Tallini G, Carlson DL, Katabi N, Shaha A, et al. Follicular variant of papillary thyroid carcinoma: a clinicopathologic study of a problematic entity. Cancer. 2006;107(6):1255-64. 10.1002/cncr.22138 16900519

[4] Shi X, Liu R, Basolo F, Giannini R, Shen X, Teng D, et al. Differential Clinicopathological Risk and Prognosis of Major Papillary Thyroid Cancer Variants. J Clin Endocrinol Metab. 2016;101(1):264-74. 10.1210/jc.2015-2917 PMC4701842 26529630

[5] Rivera M, Ricarte-Filho J, Knauf J, Shaha A, Tuttle M, Fagin JA, et al. Molecular genotyping of papillary thyroid carcinoma follicular variant according to its histological subtypes (encapsulated vs infiltrative) reveals distinct BRAF and RAS mutation patterns. Mod Pathol. 2010;23(9):1191-200. 10.1038/modpathol.2010.112 PMC4573468 20526288

[6] Vivero M, Kraft S, Barletta JA. Risk stratification of follicular variant of papillary thyroid carcinoma. Thyroid. 2013;23(3):273-9. 10.1089/thy.2012.0369 23025507

[7] Nikiforov YE, Seethala RR, Tallini G, Baloch ZW, Basolo F, Thompson LDR, et al. Nomenclature Revision for Encapsulated Follicular Variant of Papillary Thyroid Carcinoma: A Paradigm Shift to Reduce Overtreatment of Indolent Tumors. JAMA Oncol. 2016;2(8):1023-9. 10.1001/jamaoncol.2016.0386 PMC5539411 27078145

[8] Lloyd RV, Osamura RY, Klöppel G, Rosai J. WHO Classification of Tumors of Endocrine Organs. 4th ed. Lyon: IARC Press; 2017. 355p.

[9] Poller DN, Nikiforov YE. Non-invasive Follicular Thyroid Neoplasm With Papillary-Like Nuclei: Reducing Overtreatment by Reclassifying an Indolent Variant of Papillary Thyroid Cancer. J Clin Pathol. 2016;69:947-8. 10.1136/jclinpath-2016-203930 27387983

[10] Valdebarrano P, Khazai L, Thompson ZJ, Sharpe SC, Tarasova VD, Otto KJ, et al. Cancer Risk Associated with Nuclear Atypia in Cytologically Indeterminate Thyroid Nodules: A Systematic Review and Meta-Analysis. Thyroid. 2018;28(2):210-9. 10.1089/thy.2017.0419 PMC7869885 29160163

[11] Murata SI, Mochizuki K, Nakazawa T, Kondo T, Nakamura N, Yamashita H, et al. Detection of underlying characteristics of nuclear chromatin patterns of thyroid tumor cells using texture and factor analyses. Cytometry. 2002;49(3):91-5. 10.1002/cyto.10162 12442308

[12] Wang W, Ozolek JA, Rohde GK. Detection and Classification of Thyroid Follicular Lesions Based on Nuclear Structure from Histopathology Images. Cytometry. 2010;77(5):485-94. 10.1002/cyto.a.20853 PMC3010854 20099247

[13] Jung C, Kim C. Impact of the accuracy of automatic segmentation of cell nuclei clusters on classification of thyroid follicular lesions. Cytometry A. 2014;85(8):709-18. 10.1002/cyto.a.22467 24677732

[14] Valentim FO, Coelho BP, Miot HA, Hayashi CY, Jaune DTA, Oliveira CC, et al. Follicular thyroid lesions: is there a discriminatory potential in the computerized nuclear analysis? Endocr Connect. 2018;7(8):907-13. 10.1530/EC-18-0237 PMC6063880 29973373

[15] ImageJ. Avaliable at: http://imagej.net/Downloads. Accessed in: 2 Mar. 2015.

[16] Crissman JD, Drozdowicz S, Johnson C, Kini SR. Fine needle aspiration diagnosis of hyperplastic and neoplastic follicular nodules of the thyroid. A morphometric study. Anal Quant Cytol Histol. 1991;13(5):321-8. 1801830

[17] Miot HA. Assessing normality of data in clinical and experimental trials. J Vasc Bras. 2017;16:88-91.

[18] Song YS, Won JK, Yoo SK, Jung KC, Kim MJ, Kim SJ, et al. Comprehensive Transcriptomic and Genomic Profiling of Subtypes of Follicular Variant of Papillary Thyroid Carcinoma. Thyroid. 2018;28(11):1468-78. 10.1089/thy.2018.0198 30226444

[19] Maletta F, Massa F, Torregrossa L, Duregon E, Casadei GP, Basolo F, et al. Cytological features of “noninvasive follicular thyroid neoplasm with papillary-like nuclear features” and their correlation with tumor histology. Hum Pathol. 2016;54:134-42. 10.1016/j.humpath.2016.03.014 27085556

[20] Sohn SY, Lee JJ, Lee JH. Molecular Profile and Clinicopathologic Features of Follicular Variant Papillary Thyroid Carcinoma. Pathol Oncol Res. 2020;26(2):927-36. 10.1007/s12253-019-00639-8 30900082

